# Quadriceps-Bone-Block Autograft Anterior Cruciate Ligament Reconstruction With Tibial Interference Screw Aperture Fixation and Suture Tape Reinforcement

**DOI:** 10.1016/j.eats.2025.103984

**Published:** 2025-10-31

**Authors:** Taylor M. Ricci, Alexander J. Hoffer

**Affiliations:** University of British Columbia Department of Orthopedic Surgery, Gordon and Leslie Diamond Health Care Centre, Vancouver, Canada

## Abstract

Anterior cruciate ligament (ACL) reconstruction is one of the most common orthopaedic surgical procedures. The risk of clinical failure after isolated ACL reconstruction in high-risk populations is as high as 40% at 2 years postoperatively. Multiple strategies exist to decrease the risk of rerupture after ACL reconstruction, including manipulation of the graft source and diameter, optimization of the femoral and tibial tunnel position, addition of concomitant extra-articular procedures, and graft support with synthetic material. Independent graft reinforcement with a high-strength, nonabsorbable suture tape has become a popular, low-cost, low-morbidity technique to increase the graft yield strength, decrease graft stretching, improve clinical outcomes, and decrease rerupture rates. However, most suture tape reinforcement techniques require suspensory fixation of both the femoral and tibial sides, with no alternative for bone-block aperture fixation. Furthermore, the existing techniques for independent suture tape reinforcement with tibial aperture fixation are overly complex, requiring intra-articular shuttling of thick, multi-braided suture limbs. We present a simple, extra-articular shuttling technique for independent suture reinforcement of quadriceps-bone-block autograft ACL reconstruction with tibial interference screw aperture fixation.

The anterior cruciate ligament (ACL) is the most injured knee ligament, and in turn, ACL reconstruction is a commonly performed orthopaedic surgical procedure.^1^^,^^2^ Recurrent instability after ACL rupture increases the risk of subsequent meniscal tear, chondral injury, and progression to osteoarthritis.^3^ Furthermore, despite extensive research dedicated to improving outcomes after ACL reconstruction, the rerupture rate remains high.^4^^,^^5^ Optimal tunnel position, graft selection, and augmentation strategies—including lateral extra-articular tenodesis and suture tape reinforcement—are important areas of innovation that may improve postoperative outcomes. The Internal Brace (Arthrex, Naples, FL) is a type of suture tape made up of 2 strands of high-strength, nonabsorbable ultra-high-molecular-weight polyethylene and polyester weave, designed to enhance graft stability with minimal additional morbidity or cost.^3^ The Internal Brace may be tensioned independently of the graft, termed “reinforcement,” or tensioned simultaneously, termed “augmentation.” Early evidence suggests that suture tape reinforcement of an ACL reconstruction results in lower graft stress shielding while decreasing the rate of rerupture compared with an isolated ACL reconstruction.^6^^,^^7^

Previous studies have described independent suture tape reinforcement of hamstring, bone-patellar tendon-bone, and all-soft-tissue quadriceps autograft ACL reconstruction.^8^^,^^9^ This article presents a technique for suture tape reinforcement of a quadriceps-bone-block autograft ACL reconstruction with tibial interference screw aperture fixation.

## Surgical Technique

### Preoperative Workup

The workup after an ACL rupture includes a detailed history, physical examination, and imaging including radiographs and magnetic resonance imaging. If clinical symptoms of rotational instability stop the patient from returning to desired activities despite nonoperative optimization through physical therapy and bracing, surgical reconstruction is indicated.

### Surgical Positioning

After a general anesthetic is administered, an examination under anesthesia is completed. The patient is positioned supine with a side post and foot positioner. A tourniquet is applied to the operative thigh. Anatomic landmarks are outlined on the skin for the knee arthroscopy portals, graft harvest, and tunnel formation (Video 1).

### Quadriceps Graft Harvest and Preparation

**Fig 1:**
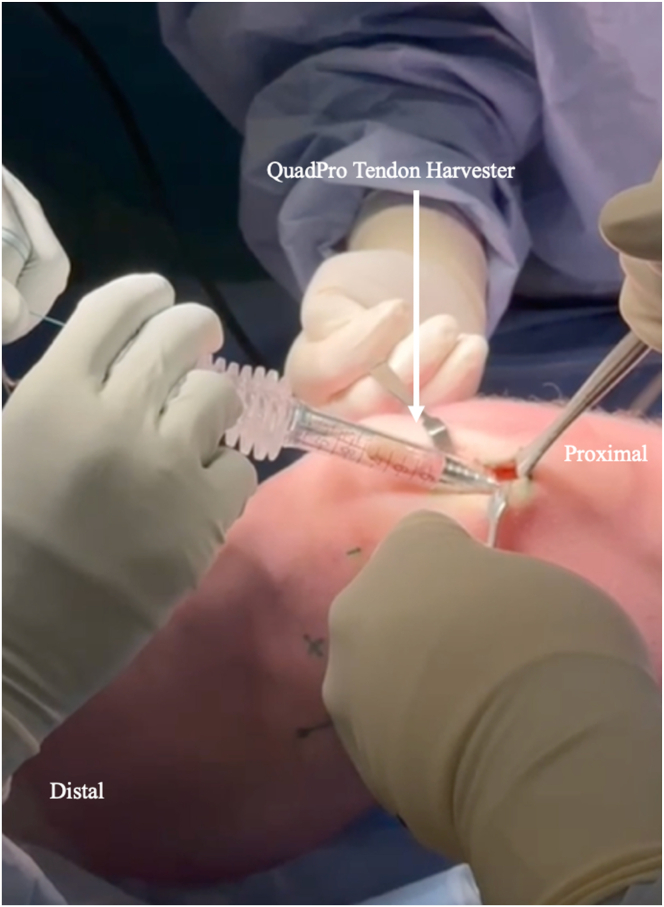
The QuadPro tendon harvester is used to harvest an 85- to 90-mm graft from the central third of the quadriceps tendon. This is performed with the patient in the supine position.

A 5-cm midline longitudinal incision is centered at the insertion of the quadriceps tendon at the proximal pole of the patella. A triangular 10-mm-wide, 20-mm-long patellar bone block is harvested with an oscillating saw distal to the central third of the tendon after a 2-mm drill hole is made in the middle of the distal third of the bone block. A QuadPro tendon harvester (Arthrex) is used to harvest 65 to 70 mm of the central third of the tendon to obtain an 85- to 90-mm graft (Fig 1, Video 1).

**Fig 2:**
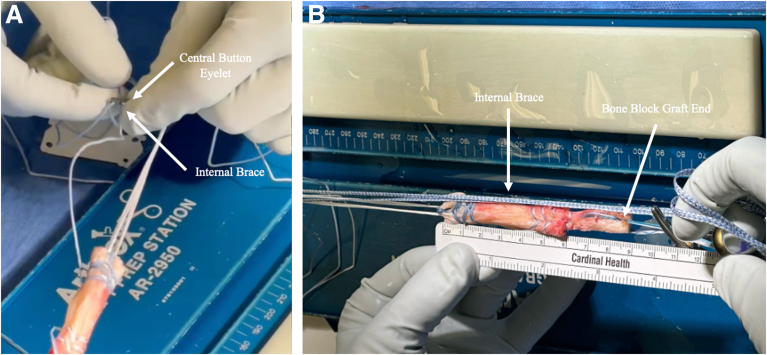
(A) The Internal Brace is added through the central button eyelet using a straight needle with a looped end. (B) Final graft with No. 2 FiberWire sutures and Internal Brace with bone block.

Two No. 2 FiberWire sutures (Arthrex) are passed through the 2-mm drill hole, through the Sharpey fibers, and back through the central hole for control, and a third No. 2 FiberWire is whipstitched up and down the distal tendon as a safety suture. A FiberTag TightRope (Arthrex) is placed on the tendon end in preparation for femoral-sided suspensory fixation. An Internal Brace is added through the central button eyelet, and the graft is placed under 80 N of tension and wrapped in vancomycin-soaked sponge until graft insertion (Fig 2, Video 1).

### Knee Arthroscopy and Tunnel Formation

Standard arthroscopic portals are established, and diagnostic knee arthroscopy is completed. Any associated intra-articular pathology is treated appropriately. A femoral socket is made with an outside-in technique using a FlipCutter (Arthrex) to a depth of 25 mm, with at least a 7-mm outer cortical width. A 35- to 40-mm open tibial tunnel is drilled outside-in with a coring reamer (Arthrex) to obtain a bone dowel for patellar grafting (Video 1).

### Femoral Graft Fixation

The lead passing sutures, FiberTag button, and looped Internal Brace suture tape limbs are shuttled through the tibial tunnel into the femoral tunnel. The button is flipped on the outer femoral cortex, and the graft is seated in the femoral tunnel via the shortening mechanism of the adjustable loop to a depth of 20 mm. The knee is cycled to remove creep before tibial fixation. The bone-block position in the tibial tunnel is evaluated in full extension for graft-tunnel mismatch. If the bone block remains prominent, the graft may be bottomed out in the femoral socket (Video 1).

### Tibial Graft Fixation and Suture Tape Reinforcement

**Fig 3:**
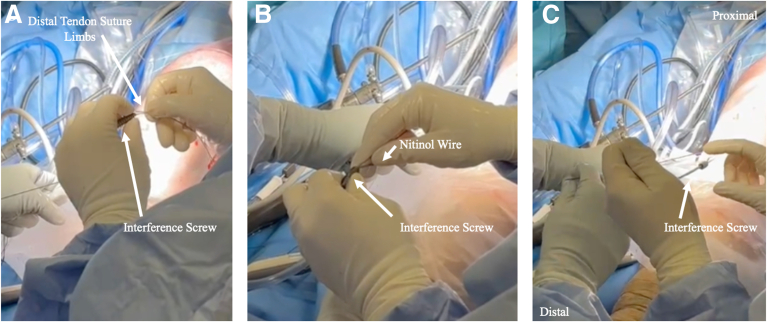
Preparation of interference screw. (A) The distal tendon suture limbs are passed through cannulated metal interference screw (Arthrex). (B) After the suture limbs are passed, the nitinol wire—which has already been inserted in the tibial tunnel adjacent to the bone block and distal quadriceps tendon—is passed through the metal interference screw. (C) The screw is passed down to the level of bone over the distal suture limbs and the nitinol wire.

**Fig 4:**
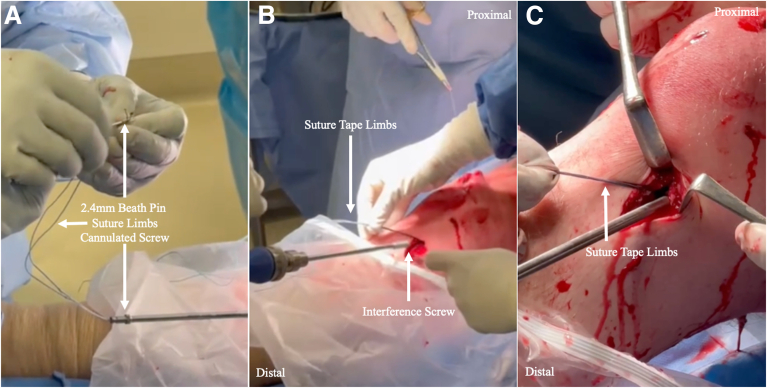
The suture limbs are shuttled through a cannulated screwdriver (Arthrex). (A) A reversed 2.4-mm Beath pin is used to shuttle the suture limbs through the screwdriver handle. (B) The screw is inserted over the nitinol wire to achieve aperture press-fit fixation. (C) The suture tape limbs should slide and remain unfixed after placement of the interference screw.

The suture tape suture ends are separated from the bone-block and distal tendon suture limbs and passed through a cannulated metal interference screw (Arthrex). The limbs are shuttled through a cannulated screwdriver (Arthrex) using a reversed 2.6-mm Beath pin (Fig 3). A nitinol wire is inserted into the tibial tunnel adjacent to the bone block and distal quadriceps tendon, with care to avoid an intra-graft wire position. The wire position is confirmed arthroscopically. Aperture press-fit fixation is achieved with the interference screw at 15° to 30° of knee flexion and neutral rotation while maintaining maximum tension on the graft sutures (Fig 4). The suture tape limbs should slide and remain unfixed after placement of the interference screw. Suture tape independence can be confirmed via arthroscopic intra-articular assessment and probing of the suture limbs after the tibial interference screw is fixed, while the graft is inspected (Video 1).

**Fig 5:**
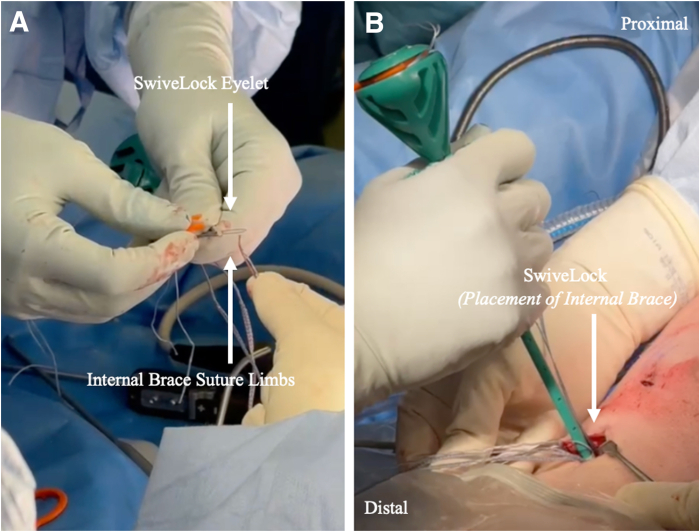
Independent suture tape fixation. (A) A 4.75-mm closed-eyelet SwiveLock suture anchor is loaded with the suture augmentation limbs. (B) The anchor is deployed in the usual fashion distal to the tibial interference screw with the knee in maximum extension.

Independent suture tape fixation is achieved with a 4.75-mm closed-eyelet SwiveLock suture anchor (Arthrex) distal to the tibial interference in full knee extension or hyperextension under manual tension. The suture tape reinforcement limbs should appear slightly loose under arthroscopic assessment in 90° of flexion after final fixation owing to the non-isometry of the ACL (Fig 5, Video 1).

### Rehabilitation Protocol

The rehabilitation protocol is dictated by the concomitant procedures completed. If an isolated quadriceps–bone-block ACL reconstruction with suture tape reinforcement is performed, routine rehabilitation is initiated immediately after surgery, including full weight-bearing status, progressive range of motion, and physiotherapy with objective performance measures used to indicate return to sport at approximately 9 to 12 months postoperatively (Video 1).

## Discussion

Suture tape augmentation and reinforcement have improved both biomechanical characteristics and clinical outcomes after ACL reconstruction in previous studies. Bachmaier et al.^6^ showed that suture tape augmentation prevented graft elongation and decreased graft loads during the healing and maturation period. However, independent reinforcement may have an advantage over simultaneous tensioning in suture augmentation by avoiding graft stress shielding while maintaining increased yield strength and maximum load to failure.^6^ Furthermore, Daniel et al.^10^ showed that the addition of suture tape reinforcement may decrease the risk of revision ACL reconstruction while maintaining comparable patient-reported outcomes when applied in an appropriate clinical scenario. The ability to achieve independent suture reinforcement of high–tensile strength quadriceps autograft and bony press-fit tibial fixation during ACL reconstruction provides several major theoretical advantages that may contribute to superior clinical outcomes in high-risk populations.

This report describes the rationale, technique, and limitations of suture tape reinforcement of a quadriceps–bone-block autograft ACL reconstruction with aperture tibial interference screw fixation (Table 1). Benefits include independent graft reinforcement with a high-strength, nonabsorbable suture while maintaining press-fit, aperture bone-to-bone fixation within the tibial tunnel (Table 2). Limitations include the initial learning curve and possible over-constraint of the knee if the suture tape is tensioned incorrectly. Clinical studies are necessary to assess the clinical outcomes and complications of the reported technique compared with conventional isolated ACL reconstruction techniques.

**Table 1:** Pearls and Pitfalls of Suture Tape Reinforcement With Tibial Aperture Interference Screw Technique

Surgical Step	Pearls	Pitfalls
Suture tape addition to ACL FiberTag TightRope	Ensure the suture tape limbs are separate from the graft TightRope sutures.	There is potential for tangling of the suture tape limbs and TightRope, which may compromise adjustable loop suture mechanism.
ACL graft tibial aperture interference screw fixation	Place the suture tape limbs through a 2.6-mm Beath pin to shuttle them through a cannulated screwdriver. Arthroscopically confirm the nitinol wire position adjacent to but not within the graft. Maintain the tension of the suture tape while placing the screw and screwdriver over the nitinol wire.	Attempting to pass the suture tape ends through a screwdriver without a reversed 2.6-mm Beath pin is onerous and difficult. Screw fixation when the nitinol wire is within the graft can compromise graft integrity. Bundled suture tape can become caught between the interference screw and tunnel wall, resulting in premature suture tape fixation with inappropriate tension.
Suture tape tibial fixation	Tension and fix in full knee extension or hyperextension if present. Tap before SwiveLock suture anchor insertion to facilitate complete anchor placement.	There is a risk of knee over-constraint and loss of full extension if the suture tape is fixed in flexion. There is potential for incomplete SwiveLock engagement or seating in the tibia if the bone is not tapped because of hard proximal tibial bone.

ACL, anterior cruciate ligament.

**Table 2:** Advantages and Disadvantages of Common Strategies for Suture Tape Addition to ACL Reconstruction

	Reinforcement	Augmentation
Suspensory tibial fixation	Advantages: Original description of suture tape reinforcement necessitated suspensory tibial fixation with an extracortical button Disadvantages: Bailout if graft-socket mismatch and “bottoming out” requires socket alteration	Advantages: Original description of suture tape reinforcement necessitated suspensory tibial fixation with an extracortical button Disadvantages: Simultaneous fixation of the graft and suture tape results in graft stress shielding and potential over-constraint
Aperture tibial fixation	Advantages: Allows bone-bone healing with press-fit interference screw fixation and independent fixation of the graft and suture tape augmentation Disadvantages: Not previously described for quadriceps–bone-block fixation; other fixation techniques are overly complicated and require intra-articular shuttling of thick, multi-braided sutures	Advantages: Technically easier to simultaneously tension graft and suture tapes with interference screw fixation Disadvantages: Simultaneous fixation of the graft and suture tape results in graft stress shielding and potential over-constraint

ACL, anterior cruciate ligament.

## Disclosures

The authors declare the following financial interests/personal relationships which may be considered as potential competing interests: A.J.H. reports a consulting or advisory relationship with Arthrex and Smith & Nephew. The other author (T.M.R.) declares that they have no known competing financial interests or personal relationships that could have appeared to influence the work reported in this paper.

## Supplementary Data

Video 1 Step-by-step technical video outlining the proposed technique to perform a quadriceps–bone-block autograft anterior cruciate ligament reconstruction with tibial interference screw aperture fixation and suture tape reinforcement.
